# Clinical and Imaging Phenotype of Schwannomatosis in Children, Adolescents and Young Adults

**DOI:** 10.3390/cancers18162701

**Published:** 2026-08-20

**Authors:** Shivani Ahlawat, Jaishri O. Blakeley, Laura M. Fayad, Krista S. Schatz, Stephanie M. Morris, Allan J. Belzberg, Bronwyn Slobogean, Carlos G. Romo

**Affiliations:** 1 The Russel H. Morgan Department of Radiology and Radiological Science, Johns Hopkins University School of Medicine, Johns Hopkins Medical Institutions, Baltimore, MD 21287, USA; lfayad1@jhmi.edu; 2 Department of Neurology, Neuro-Oncology, Johns Hopkins University School of Medicine, Baltimore, MD 21287, USA; jblakel3@jhmi.edu (J.O.B.);; 3 Department of Orthopaedic Surgery, The Johns Hopkins Medical Institutions, Baltimore, MD 21287, USA; 4 Department of Oncology, The Johns Hopkins Medical Institutions, Baltimore, MD 21287, USA; 5 Department of Genetic Medicine, Johns Hopkins University School of Medicine, Baltimore, MD 21287, USA; ksonder1@jhmi.edu; 6 Department of Neurology and Neurogenetics, Kennedy Krieger Institute, Baltimore, MD 21205, USA; smorri94@jhmi.edu; 7 Department of Neurosurgery, Johns Hopkins University School of Medicine, Baltimore, MD 21287, USA; abelzbe1@jhmi.edu (A.J.B.); bslobog1@jhmi.edu (B.S.)

**Keywords:** schwannomatosis, neurofibromatosis type 2, schwannoma, meningioma, ependymoma, magnetic resonance imaging

## Abstract

**Background/Objectives:** To characterize the clinical and imaging phenotypes of schwannomatosis (SWN) in children, adolescents, and young adults (AYAs). **Methods:** This single-center retrospective cohort study included patients (age ≤ 25 years) with suspected SWN with archived magnetic resonance imaging (MRI) at Johns Hopkins between 2020–2026. Clinical characteristics, genetic testing results, and tumor imaging (including anatomic distribution), and treatment approaches were reviewed. Descriptive statistics were performed. **Results:** In total, thirty-eight patients with suspected SWN were identified (median age 20 years, range 6–25; 32% female). *NF2*-related SWN was the most common diagnosis (31/38, 82%; de novo: 24/31 (77%) and familial: 7/31 (23%)), followed by *LZTR1*-SWN (*n* = 1) and SWN-NOS/NEC (*n* = 2). A total of four patients (asymptomatic *LZTR1* carriers (*n* = 2) and possible SWN (*n* = 2)) did not fulfill diagnostic criteria based on craniospinal and whole-body MRI. Familial *NF2*-SWN demonstrated a trend toward earlier presentation (median 5 vs. 8 years; *p* = 0.12). Visual manifestations occurred at a younger age than hearing loss (median 8 vs. 16 years; *p* = 0.053). Imaging burden was high (median 23 MRI/patient (range: 1–60). Bilateral vestibular schwannomas were nearly universal (30/31, 97%). Familial disease demonstrated a trend toward increased cranial meningiomas (86% vs. 54%; *p* = 0.20), while peripheral schwannomas (43% vs. 67%; *p* = 0.38) and ependymomas (14% vs. 42%; *p* = 0.37) were less frequent compared with de novo disease. Operative and non-operative management was documented in 65% (20/31) each. **Conclusions:** *NF2*-related SWN is the predominant subtype in a pediatric and AYA patient population and was associated with substantial early tumor burden at a tertiary specialty NF center.

## Simple Summary

Schwannomatosis (SWN), an umbrella term for a group of rare genetic conditions, causes schwannomas to grow along nerves throughout the body. This study examined imaging findings in 38 children, adolescents and young adults (≤25 years) with suspected SWN to better understand how SWN presents early in life. The majority in this cohort had *NF2*-related SWN (82%) and were diagnosed at around 9 years of age. MRI of central and peripheral nervous system revealed bilateral vestibular scwhannomas, meningioma and ependymoma in many but not all patients. Many patients required surgery, while others received systemic therapy. Diagnosing SWN in children and young adults can be challenging, requiring multidisciplinary assessment. The study highlights the importance of advanced imaging (including whole-body MRI) for detecting tumors throughout the body and genetic testing to guide treatment decisions and improve care for children and young adults with SWN.

## 1. Introduction

Recent updates in the diagnostic criteria and nomenclature for neurofibromatosis type 2 (NF2) and schwannomatosis (SWN) incorporate both clinical and molecular features to distinguish *NF2*-related schwannomatosis *(NF2*-SWN) from non-*NF2*-SWN, including *SMARCB1*-, *LZTR1*-, and 22q-SWN [1]. The terms SWN-NOS (not otherwise specified) or SWN-NEC (not elsewhere classified) refer to individuals who meet the clinical criteria of multiple confirmed schwannomas across more than one nerve distribution but without definitive molecular classification due to absent or non-diagnostic genetic testing, respectively [1]. Based on the UK National NF2 database, *NF2*-SWN is the most common subtype (estimated prevalence of ~1/61,000) followed by *LZTR1*-SWN (~ 1/527,000) and *SMARCB1*-SWN (~1/1.1 million) [2].

Diagnosis of SWN in pediatric populations remains particularly challenging because characteristic tumor burden and classic phenotypic manifestations evolve over time, and younger patients may not yet meet established clinical criteria. In addition, mosaicism is present in up to half of people with de novo *NF2*-related SWN, and germline testing is often non-diagnostic in the setting of mosaicism [1,3,4]. Furthermore, *LZTR1*- and 22q-SWN can frequently have reduced penetrance, segmental tumor distributions, and variable expressivity—also complicating definitive diagnosis [5]. Accordingly, current diagnostic guidelines recommend molecular analysis of non-tumor tissue, such as blood or saliva, in addition to ≥2 anatomically distinct tumors when feasible to reach a definitive diagnosis of SWN [1,4].

*NF2*-SWN is classically associated with bilateral vestibular schwannomas (VSs), non-VS intracranial and extracranial (peripheral nerve and paraspinal) schwannomas, intradermal schwannomas, ependymomas, meningiomas as well as ophthalmologic manifestations such as retinal hamartomas or juvenile cataracts [1]. Furthermore, *NF2* pathogenic variant type correlates strongly with disease severity and age at presentation, with constitutional truncating mutations—particularly involving exons 2–13—associated with earlier onset, greater tumor burden, more aggressive disease, and worse hearing outcomes [6,7,8,9]. In contrast, missense, splice-associated, mosaic, and large deletion variants generally exhibit milder or more variable phenotypes with later clinical presentation and slower progression [6,8].

In contrast, non-*NF2*-SWN subtypes are more commonly associated with multiple non-intradermal schwannomas, with meningiomas reported more often in *SMARCB1*-SWN and unilateral VS occurring in some cases of *LZTR1*-SWN [2,3,6]. Unfortunately, relatively little is known regarding the burden, distribution, or biology of tumors in younger patients with non-*NF2*-SWN [10,11]. The existing literature regarding genotype–phenotype correlations, natural history, surveillance recommendations, and imaging findings is derived predominantly from adult cohorts, with pediatric and adolescent populations remaining underrepresented, particularly among those with the non-*NF2*-SWN subtype [9,10,12,13,14,15,16,17,18,19]. Early recognition of imaging patterns in children and adolescents may facilitate more timely diagnosis, reduce diagnostic uncertainty, improve anticipatory counseling for affected families and potentially define optimal patient populations for early treatments.

Accordingly, this study aimed to characterize the clinical presentation, imaging features, and management patterns of SWN in children, adolescents, and young adults (AYAs) in the setting of the recent evolution of SWN nomenclature and molecular classification systems.

## 2. Materials and Methods

This institutional review board approved HIPAA-compliant retrospective cohort study was performed at a tertiary referral center with a comprehensive neurofibromatosis (NF) clinic. The institutional electronic imaging database was queried using Slicer Dicer to identify imaging studies performed between 1 April 2020 and 20 April 2026 using the following search terms: Neurofibromatosis type 2, Neurofibromatosis II, NF2, Central NF2 neurofibromatosis, NF2 (neurofibromatosis type 2), *SMARCB1*-related schwannomatosis, *LZTR1*-related schwannomatosis, *NF2*-related schwannomatosis, schwannomatosis associated with mutation in *SMARCB1* gene, schwannomatosis associated with mutation in *LZTR1* gene, schwannomatosis associated with mutation in *NF2* gene, schwannomatosis. Inclusion criteria were age ≤ 25 years old and available imaging within the institutional electronic database [1]. Age ≤ 25 years was selected to capture pediatric and AYA presentations during periods of ongoing neurodevelopment and transition to adult care. Exclusion criteria included absence of available imaging, age > 25 years old, and absence of molecular testing in patients without bilateral VSs. All cases underwent manual chart review by a single reviewer to confirm diagnostic classification based on contemporary consensus SWN criteria and available clinical and molecular data. Historical diagnoses were retrospectively reclassified according to updated nomenclature, when appropriate. Clinical data collected included age, sex assigned at birth, age at diagnosis, clinical presentation, interval between initial presentation and ultimate diagnosis, and SWN subtype (*NF2*-, *LZTR1*-, *SMARB1*-, 22q-, SWN-NOS, SWN-NEC) [1]. Available genetic testing results, including germline and/or tumor testing, were recorded.

For patients with *NF2*-SWN and available molecular data, a genetic severity score (GSS) was assigned as follows: 1: mosaic pathogenic variants identified only in tumor tissue and not detected in blood; 2A (mild): missense variants, truncating variants in exons 1 and or 13-14, splice-site variants involving exons 7–15; 2B (moderately severe): large deletions and splice-site variants involving exons 1–6; and 3 (severe): truncating variants involving exons 2–13 [6]. For patients without definitive genetic testing, SWN-NOS was defined as greater than 2 non-intradermal schwannomas identified on imaging with histological confirmation of at least one lesion as schwannoma [1]. SWN-NEC was assigned in patients without identifiable pathogenic SWN variants following analysis of at least 2 anatomically distinct tumors and unaffected tissue (typically blood or saliva) [1].

Subsequently, the electronic health record (EHR) was reviewed for imaging type, including magnetic resonance imaging (MRI) of the brain/internal auditory canal/skull base, cervical spine, thoracic spine and lumbar spine, and localized or whole-body MRI. The imaging was evaluated by one musculoskeletal radiologist with 15 years of experience in SWN for descriptive purposes. The reviewer was blinded to the molecular data/GSS category but did know that the patient population had a diagnosis of SWN. The presence, location, and imaging characteristics of schwannomas (vestibular, non-vestibular, spinal or peripheral), meningiomas (intracranial or spinal) and ependymomas (intracranial or spinal) were recorded based on characteristic MRI features and anatomic location. Given the lack of pathological confirmation for all tumors, imaging-based definitions were used as follows: vestibular schwannoma (VS), a variably enhancing mass within the internal auditory canal; meningioma, an intracranial, extra-axial mass or intraspinal, intradural extramedullary mass with dural tail; ependymoma, an intraspinal, intradural intra-medullary expansile mass; spinal schwannoma, intra-dural extramedullary mass without a dural tail; and peripheral schwannoma, a lesion contiguous with a peripheral nerve with or without “target” sign [20,21]. Operative and non-operative treatment strategies were also recorded.

Descriptive statistics were used to summarize demographic, clinical, molecular, imaging, and treatment characteristics across the cohort. Continuous variables were summarized using medians and ranges due to the small sample size and non-normal distribution of the data. Categorical variables were summarized as frequencies and percentages. Comparisons between patients with de novo and familial *NF2*-SWN were performed using Fisher’s exact test for categorical variables and the Mann–Whitney U test for continuous variables, including age, duration to diagnosis, and MRI utilization metrics. Median age and age at presentation were compared across genetic severity score groups (2A, 2B, and 3) in patients with *NF2*-SWN using the Kruskal–Wallis test due to the small sample size and non-normal distribution of the data. Statistical significance was defined as a two-sided *p* value < 0.05.

## 3. Results

Our search identified 38 patients with suspected SWN with a median age of 20 years (range 6–25 years); 12 (32%) were female and 26 (68%) were male. In this cohort of suspected SWN, the median age at diagnosis was 9 years old (range: birth to 18 years old). Median interval from clinical presentation to confirmed diagnosis was 6 months (range: 0 to 12 years). Genetic testing was available in 32/38 patients (84%). The remaining 6/38 patients (16%) met clinical diagnostic criteria for *NF2*-SWN based on the presence of bilateral VSs but did not have molecular testing available. *NF2*-SWN represented the predominant subtype within the cohort (31/38, 82%). A total of two patients were initially clinically diagnosed as having NF1 based on café-au-lait macules on physical exam and the absence of VSs at presentation. Subsequent molecular testing established the diagnoses of NF2-SWN and LZTR1-related schwannomatosis, respectively.

### 3.1. NF2-SWN

Among 31 patients with *NF2*-SWN (Table 1), 24/31(77%) had de novo disease, while 7/31 (23%) had a familial diagnosis. There was an overall male predominance (22/31, 71%). In this cohort of *NF2*-SWN, the median age at presentation was 8 years (range: 0–18 years), although familial cases tended to present earlier, with a median age of approximately 5 years (*p* = 0.12). Time from presentation to diagnosis was generally short (median 0 years, range 0–12 years) in patients with *NF2*-SWN, though slightly longer among patients with de novo disease (median 9 months, *p* = 0.006). In total, 5/31 (16%) had genetic testing pre-symptomatically based on the requests of parents due to an affected parent. The median age of diagnosis for this cohort was 5 months (range 0–16). In this cohort, one patient had MRI of the brain and IACs pre-symptomatically that did not detect VS. No other imaging is available for this individual in our medical record.

Among 25 patients with prior molecular testing (Table 2), 19 had sufficient genetic data available for GSS classification. No patients fulfilled criteria for GSS category 1. GSS category 2A/mild (*n* = 4/19, 21%) consisted primarily of missense and other non-truncating *NF2* variants. Group 2B/moderately severe (*n* = 7/19, 37%), includes large deletions, promoter/exon 1 deletions, and splice-associated variants. Group 3/severe (*n* = 8/19; 42%) consisted predominantly of non-mosaic truncating or nonsense variants involving exons 2–13 [6]. Median age and age at presentation did not significantly differ across genetic severity score groups 2A, 2B, and 3 in patients with NF2-related SWN (*p* = 0.37 for both). The majority of the patients with de novo disease had moderately severe (2B) or severe (3) genotype compared with familial disease which tended to have 2A GSS (*p* = 0.025).

Clinical presentations were variable. Visual manifestations were present in 19% at clinical presentation with ophthalmologic findings comprised of juvenile cataracts in five, epiretinal membranes in two patients and optic nerve meningioma in one patient. Patients presenting with visual manifestations had a median age of 8 years (range: 2–15 years). Sensorineural hearing loss was reported in 16% of patients, at a median age of 16 years (range: 12–18 years). Visual manifestations occurred at a younger age than hearing loss in this cohort. The difference in age of presentation between vision and hearing changes showed a strong trend toward significance (*p* = 0.053), suggesting that visual manifestations occur earlier in childhood, while hearing loss more commonly presents during adolescence.

Less frequent neurologic manifestations included weakness and gait abnormalities (10% each), seizures (6%), and rare developmental or movement disorders/dyskinesia (3%). Vision impairment (29%) and hearing loss (45%) were common overall and occurred at similar frequencies across subgroups. Soft tissue masses were reported in 16% of patients.

Imaging burden was substantial (Table 3), with patients undergoing a median of 23 MRI studies (range 1–60) that were available in our picture archiving communications system (PACS). Brain/internal auditory canal imaging was performed most frequently (median 10 studies per patient available in our PACS), followed by spine and regional imaging. Whole-body MRI was used relatively infrequently in people with both familial and de novo NF2-SWN.

**Figure 1:**
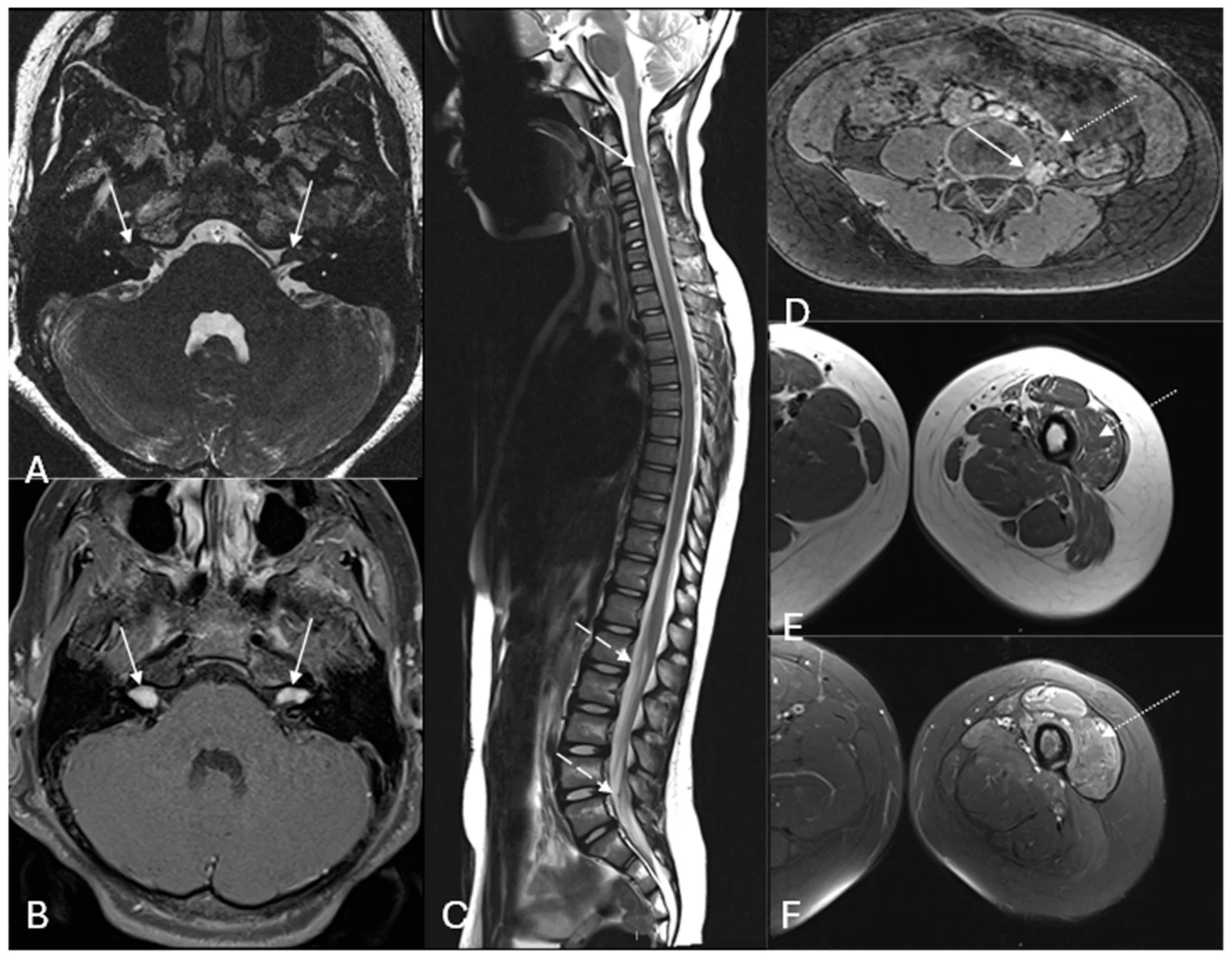
10-year-old male with de novo *NF2-related* SWN presented clinically with left lower extremity weakness and ultimate found to have bilateral VSs (arrows on (**A**,**B**)), cervical ependymoma (solid arrow on (**C**)), presumed schwannomas of the cauda equina (dashed arrows on (**C**)) and left lumbar extra-foraminal schwannoma (solid arrow on (**D**)) with resultant skeletal muscle denervation of the left psoas major (dashed arrow on (**D**)) and lower extremity including the quadriceps, adductors, sartorius and gracilis muscles (dashed arrows on (**E**,**F**)). He was ultimately treated with left femoral nerve transfers to preserve lower extremity function.

**Figure 2:**
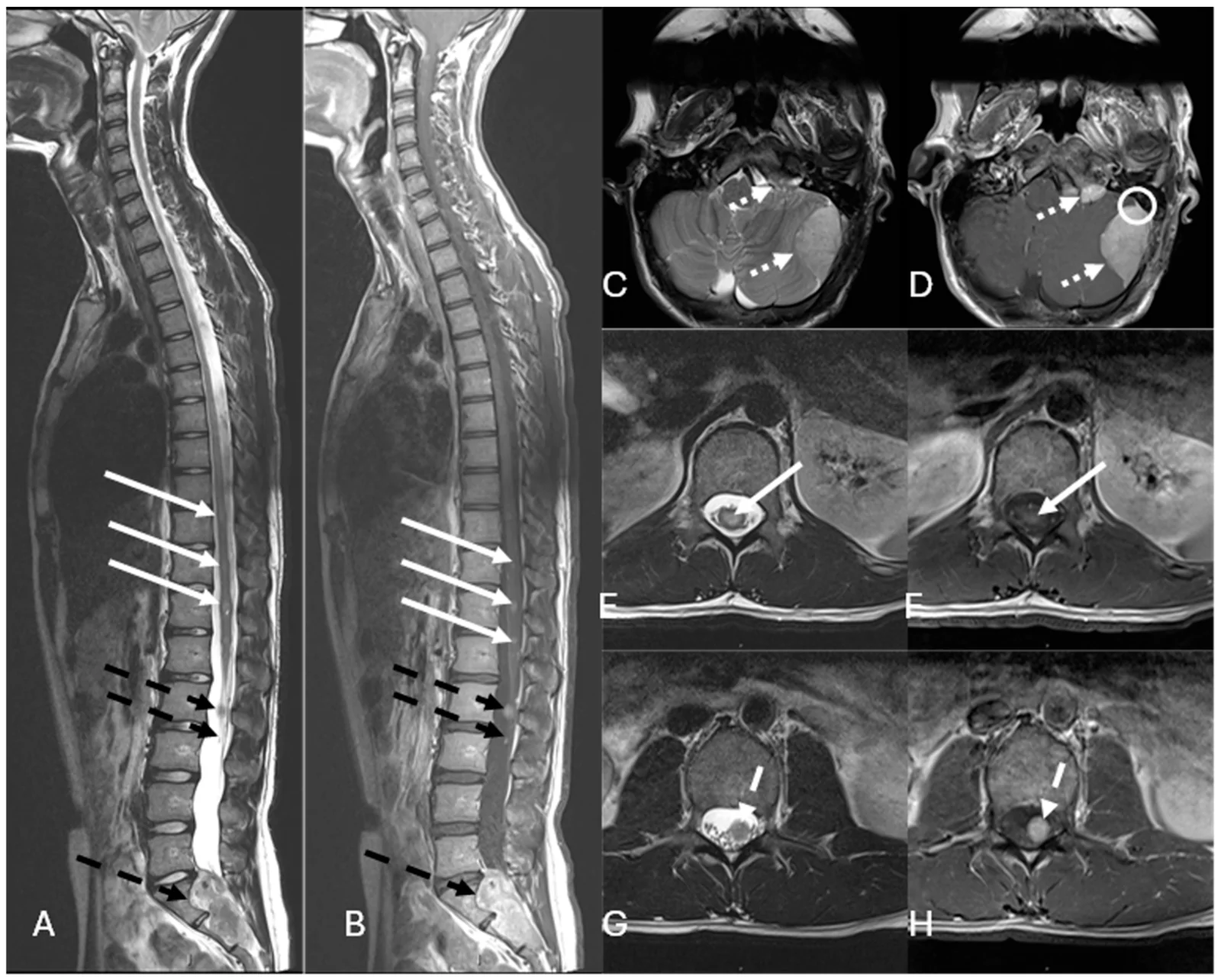
18-year-old male with de novo *NF2*-related SWN initially presented with palpable soft tissue mass after head injury ultimately found to be a schwannoma. In addition, the patient had bilateral VSs, intra-cranial meningioma (dashed white arrows on (**C**,**D**)) highlighting the dural tail (circle on (**D**)), thoracic ependymoma (white solid arrows on (**A**,**B**,**E**,**F**)) and numerous caudal equina schwannomas as well as sacral intraspinal schwannoma (dashed arrows on (**A**,**B**,**G**,**H**)).

With regard to tumor burden (Figure 1 and Figure 2), bilateral VSs were nearly universal among patients with *NF2*-SWN (97%), with similarly high frequencies in both de novo and familial disease (96% vs. 100%, *p* = 1.00). Intracranial non-VSs were only modestly higher in familial disease (86% vs. 71%; *p* = 0.64). The only intracranial lesion that clearly trended higher in familial disease was cranial meningioma (86% vs. 54%; *p* = 0.20). With regard to extra-cranial tumor burden, ependymomas (14% vs. 42%; *p* = 0.37) and peripheral schwannomas (43% vs. 67%; *p* = 0.38) were less frequent in familial disease. Among patients with available GSS, Group 3/severe variants more commonly demonstrated cranial and spinal meningiomas (*n* = 4) than Groups 2A or 2B (*n* = 1 each).

Peripheral schwannomas were the most common tumor requiring operative intervention (20 procedures in eight patients), followed by spinal schwannoma resections (11 procedures in six patients) most commonly pursued to address focal neurologic deficits. Meningioma-related surgeries were also frequent (*n* = 12), including intracranial (*n* = 4), spinal (*n* = 7), and orbital (*n* = 1) meningioma resections, with pathology ranging from WHO grade I to anaplastic WHO grade III lesions. VS debulking/resection was performed in four patients. Additional spinal tumor resections included ependymoma (*n* = 1). Tendon transfer/reconstructive procedures were performed in four patients, while peripheral nerve decompression/release procedures occurred in two patients. Radiation-based interventions included Gamma Knife radiosurgery for meningioma (*n* = 1) and thoracic intensity-modulated radiation therapy or IMRT (*n* = 1). Medical management was documented in approximately 20/31 (65%) of patients with bevacizumab (9/31, 29%) as the most frequently used treatment (Table 4).

### 3.2. LZTR1-SWN

**Figure 3:**
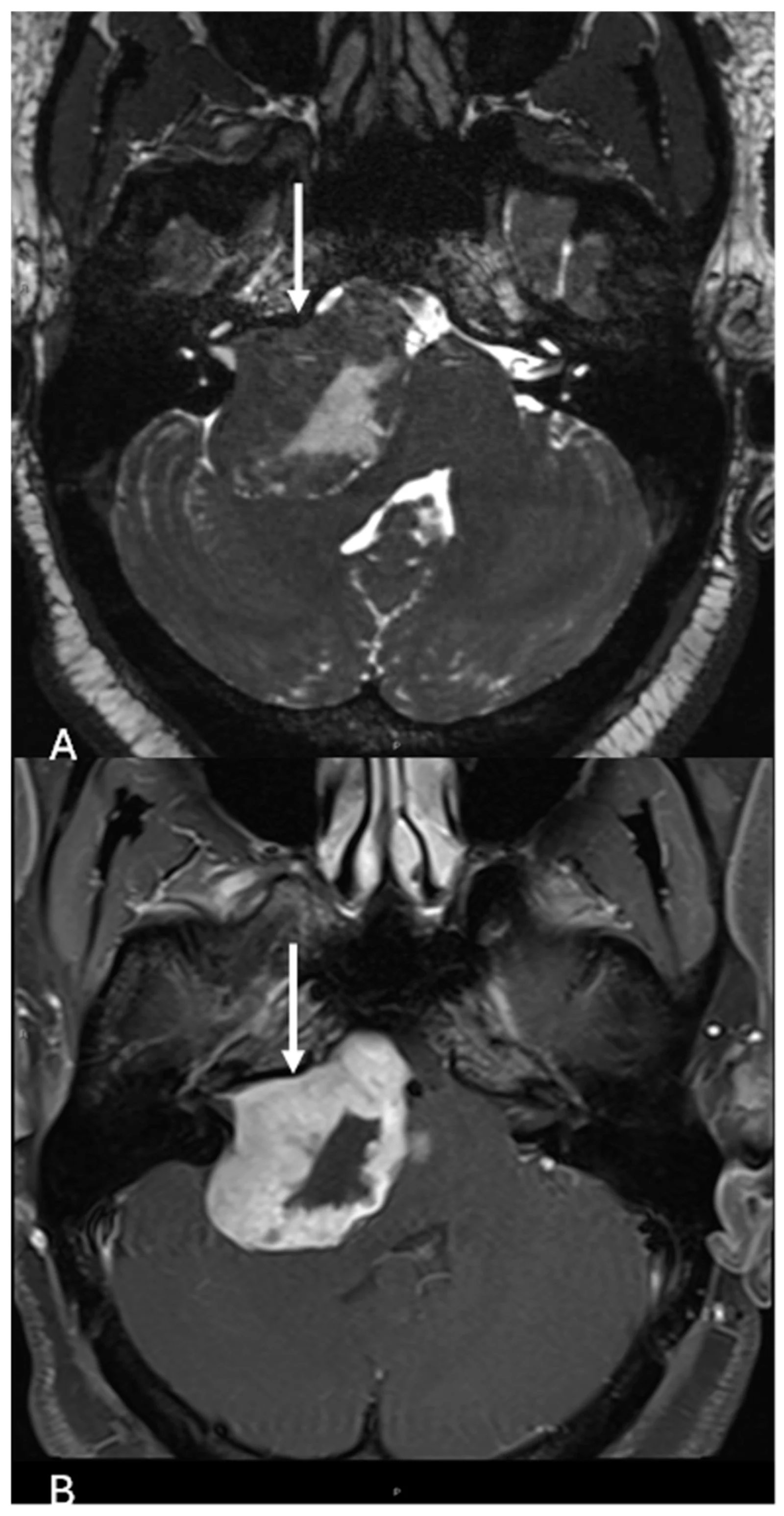
23-year-old female with *LZTR1*-related SWN and unilateral 4.7 × 3.5 × 4.5 cm^3^ VS. Axial T2 (**A**) and T1-post contrast (**B**) images through the internal auditory canals reveal a heterogeneously enhancing extra-axial mass (arrow on (**A**,**B**)) centered in the right cerebellopontine angle cistern with areas of central necrosis with widening of the porus acusticus, mass effect on the right brachium pontis and approximately 0.9 cm leftward shift at the posterior fossa.

One patient (23 years old) was diagnosed with *LZTR1*-SWN following identification of two anatomically distinct intracranial pathologically confirmed schwannomas, including one VS and one non-vestibular schwannoma (arising from right cranial nerve VI), in the setting of a likely pathogenic germline *LZTR1* variant (Figure 3).

### 3.3. SWN- NOS/NEC

**Figure 4:**
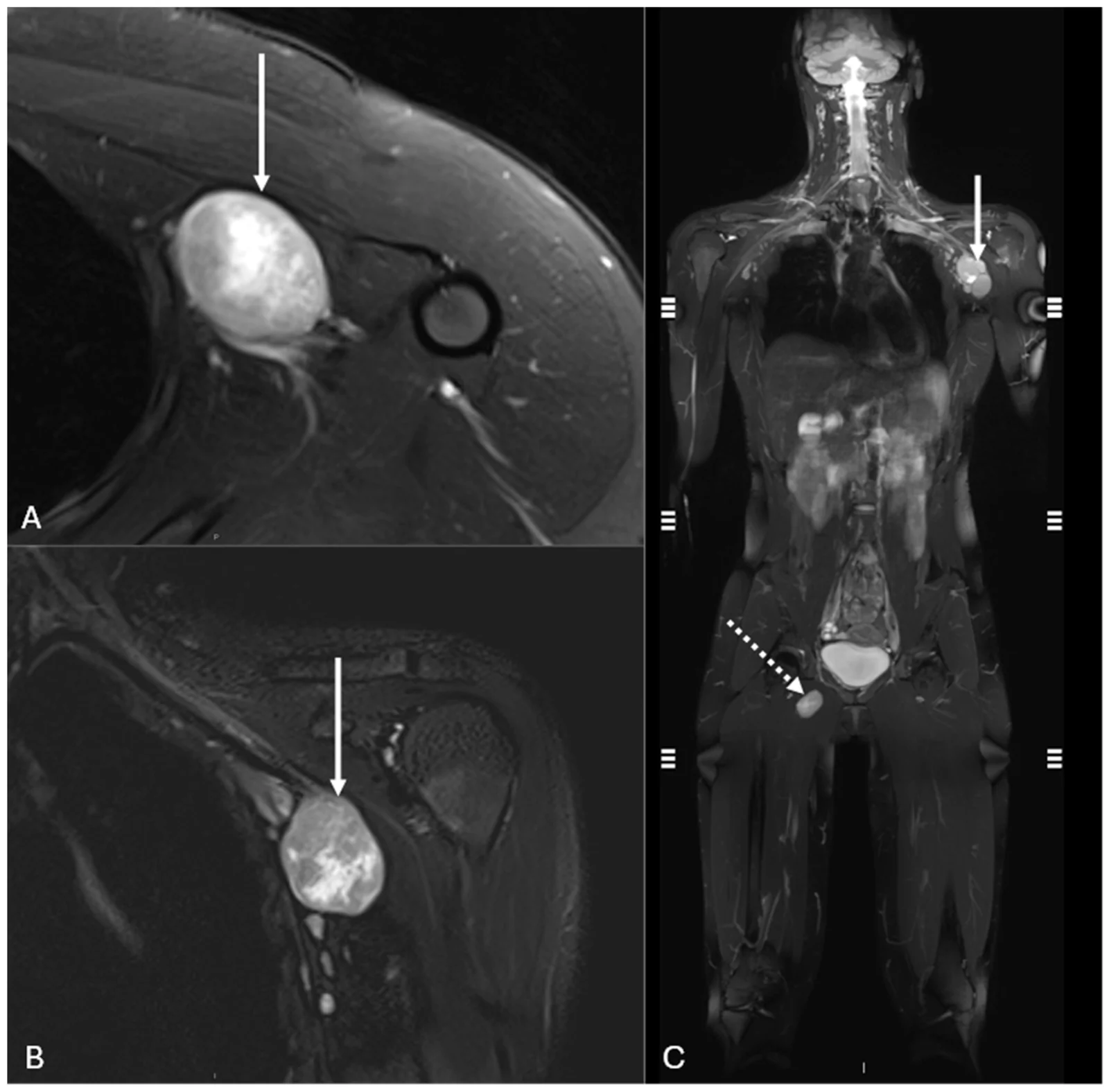
15-year-old female with positive family history for SWN-NEC and one pathologically proven left brachial plexus schwannoma (solid arrow on (**A**–**C**)) status post debulking. Whole-body MRI detected an additional right medial thigh presumed schwannoma (dashed arrow on (**C**)). Genetic testing did not detect a known pathogenic variant associated with SWN.

**Figure 5:**
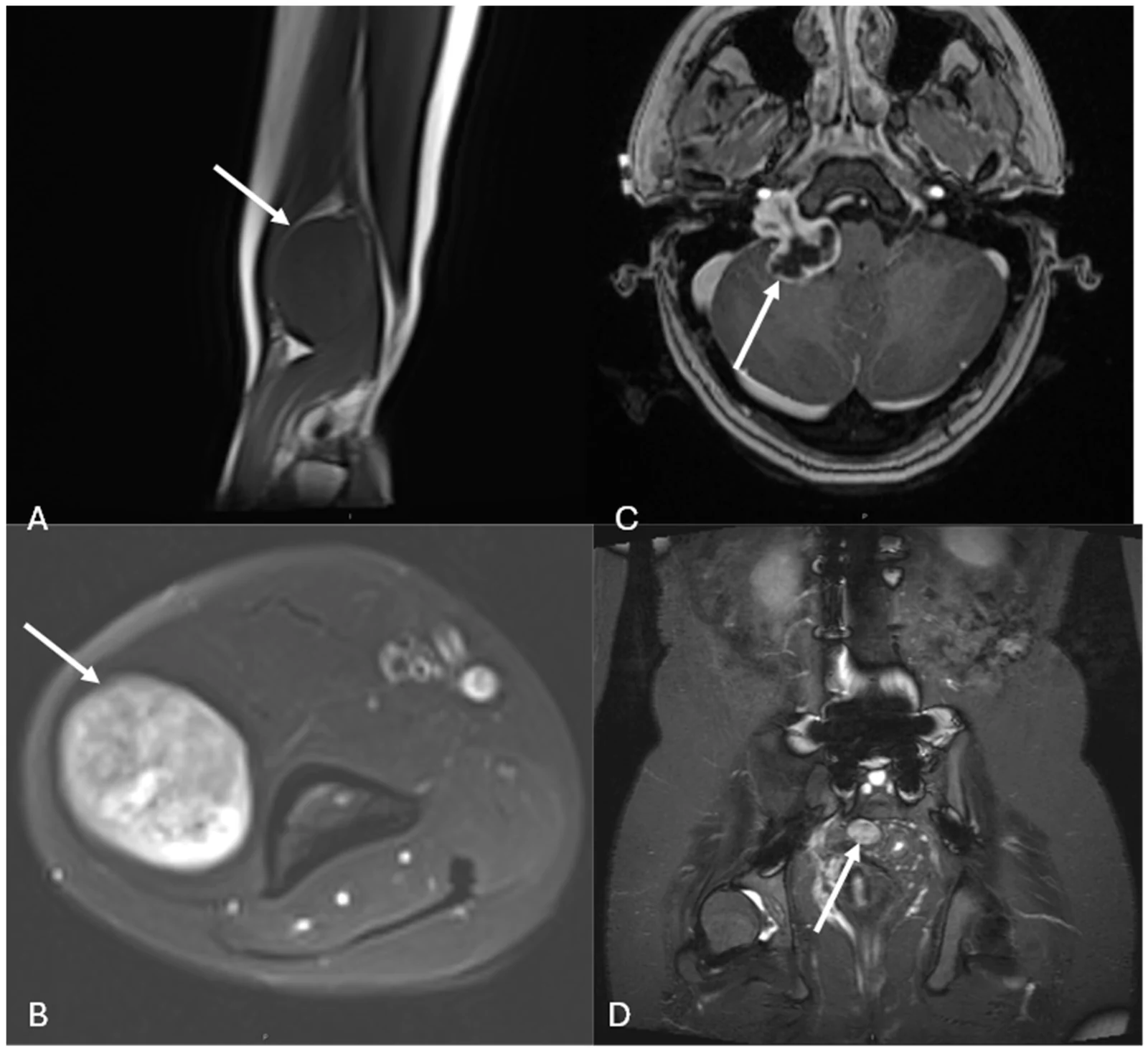
24-year-old female with pathologically proven right distal arm radial nerve schwannoma (arrow on (**A**,**B**)), right vagus nerve schwannoma (arrow on (**C**)) and a midline presacral presumed schwannoma on whole-body MRI (arrow on (**D**)). No VSs were detected on MRI of the brain and IACs. Genetic testing was not available. As such, the patient was diagnosed with SWN-NOS and managed operatively with resection of the radial nerve schwannoma and debulking of the vagus nerve schwannoma.

One 15-year-old patient (Figure 4) with positive family history of SWN, pathologically proven left distal brachial plexus schwannoma, additional presumed schwannoma on WB-MRI and negative germline testing was classified as SWN-NEC (*n* = 1). An additional patient (Figure 5) with pathologically proven right radial nerve schwannoma, additional presumed schwannomas present on brain and whole-body MRI, and unavailable genetic testing was classified as SWN-NOS (*n* = 1).

### 3.4. Diagnostically Challenging Cases Not Meeting Updated SWN Criteria

#### 3.4.1. Probable Schwannomatosis

**Figure 6:**
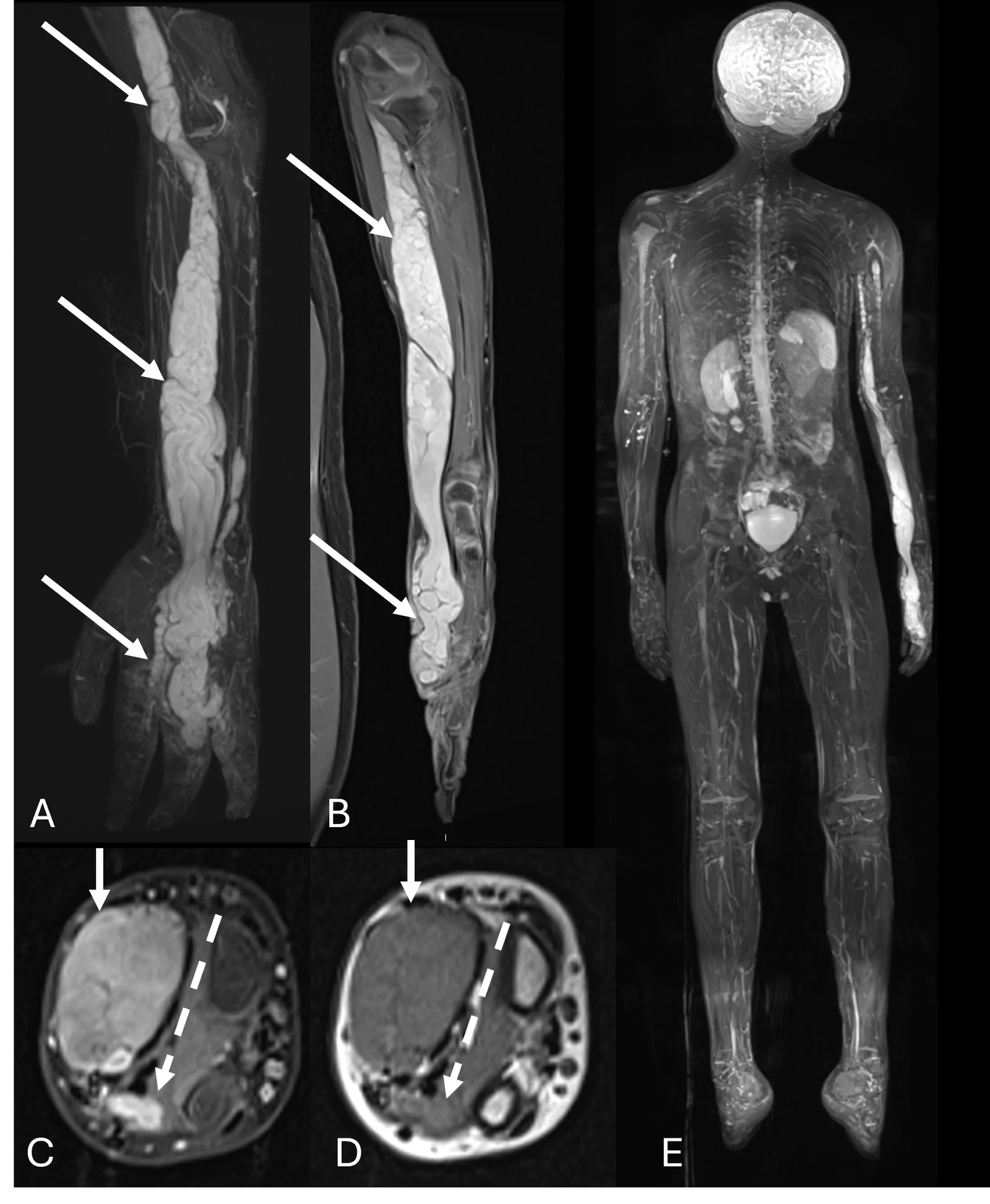
6-year-old male with left hand pain and deformity found to have a complex plexiform presumed peripheral nerve sheath tumor involving the left median (solid arrows on (**A**–**D**)) and ulnar nerves (dashed arrows on (**C**,**D**)). Genetic testing was positive for LZTR1 pathogenic variant. No other peripheral nerve tumors were detected on WB-MRI (**E**). The family declined histological confirmation due to lack of functional impairment. Currently, the patient does not meet clinical criteria for SWN due to lack of pathology proof and no other imaging or clinical features of SWN.

One patient with probable SWN initially presented at 2 years of age with a large presumed plexiform peripheral nerve sheath tumor arising from the left distal upper extremity (Figure 6). Germline testing identified a maternally inherited pathogenic LZTR1 splice donor variant. However, no additional intracranial or peripheral schwannomas were identified on brain MRI with or without contrast or whole-body MRI, and pathological confirmation was not obtained due to family preference. As such, the patient did not meet updated consensus diagnostic criteria for SWN despite clinical concern for underlying SWN disorder.

#### 3.4.2. Young Patient with Solitary Schwannoma

**Figure 7:**
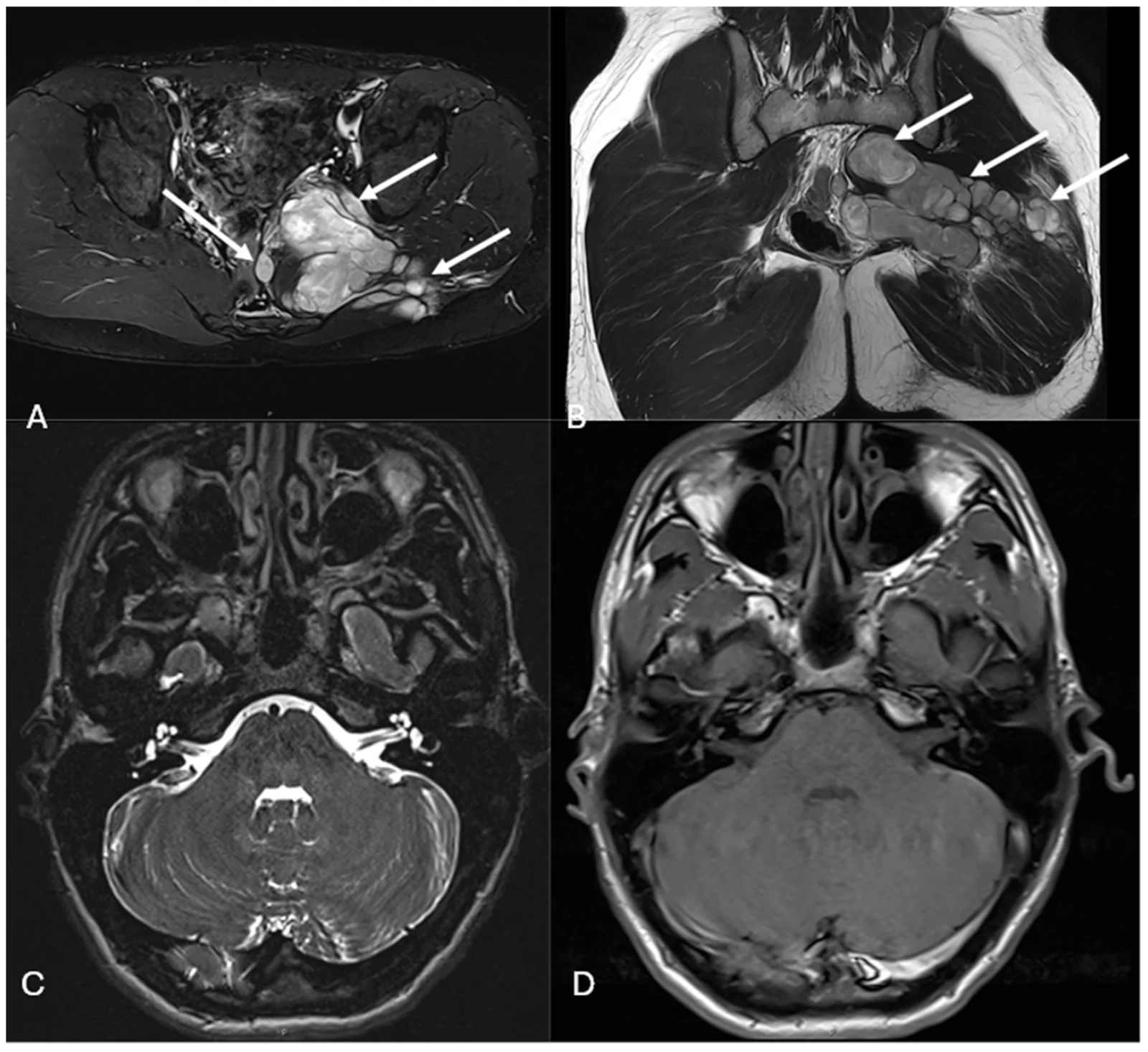
15-year-old male with complex histologically confirmed pelvis solitary schwannoma with intra- and extra-pelvic extension [arrow on axial T2-FS (**A**) and coronal T2-weighted images of the pelvis (**B**)]. Genetic testing was negative for known pathogenic variants in SWN genes as well as negative whole-body MRI and no vestibular schwannomas on MRI of the internal auditory canals (**C**,**D**). Currently, the patient does not meet clinical or genetic criteria for SWN.

A 15-year-old male patient (Figure 7) with a pathologically confirmed complex pelvic schwannoma and negative germline testing for LZTR1, NF2 and SMARCB1 did not meet diagnostic criteria for SWN. No additional intracranial, intraspinal or peripheral schwannomas were identified on brain, whole spine, and whole-body MRI.

#### 3.4.3. Asymptomatic Individuals with Germline Pathogenic Variants Associated with SWN

Within our cohort, 2/38 (5%) patients were incidentally found to harbor heterozygous pathogenic LZTR1 variants associated with SWN as well as Noonan syndrome during germline testing performed for unrelated clinical indications. Clinical indications for genetic testing included dysmorphic facial features in one patient and combination of developmental delay/short stature in the second patient. Neither patient had detectable intracranial or intraspinal lesions on MRI of the entire neuroaxis or clinically detectable peripheral schwannomas.

## 4. Discussion

In this cohort of pediatric and AYA patients with SWN, *NF2*-SWN represented the predominant subtype based on combined clinical and molecular diagnostic classification. Most cases were de novo (77%), concordant with reported high rates of non-familial *NF2*-SWN in the literature (70%) [2]. Median age at diagnosis within this cohort was much younger (9 years) than reported in predominantly adult SWN cohorts with shorter time to diagnosis (6 months). This is likely due to several factors including referral bias for more clinically severe phenotypes at a tertiary NF center, increasing use of advanced neuroimaging and molecular testing [5], and enrichment for higher-risk molecular subtypes within this cohort (78% categorized as moderately severe or severe based on GSS). Non-*NF2*-SWN remained uncommon in this younger population.

Within *NF2*-SWN, visual manifestations were common (29%). Prior pediatric studies of *NF2*-SWN have reported slightly higher frequency of vision loss in 49–66% and severe impairment associated with severe genotypes [22]. Hearing loss was also frequently present in this cohort (45%), presenting in adolescence. In addition to VSs, which were nearly universally present in this cohort, the spectrum of high tumor burden included intracranial non-VS (74%), spinal schwannomas (97%), meningiomas (61% intracranial and 29% spinal), ependymoma (36%), and peripheral schwannomas (61%) [22]. Prior pediatric *NF2*-SWN series have also reported a similar high tumor burden ranging from intracranial non-VSs (33–61%), spinal schwannomas (56%), and meningiomas (38–57% intracranial and 19% spinal), ependymomas (38%) and extra-dural schwannomas (28%) [23,24,25,26]. The relatively higher frequency of spinal and peripheral schwannomas in this cohort may be due to the higher prevalence of severe phenotypes.

One of the notable findings was the imaging burden across this cohort, with patients undergoing a median of 23 MRI exams that have been archived in our PACS per patient. Brain/internal auditory and spinal imaging predominated, reflecting the high prevalence of intracranial and intraspinal tumor burden in children and AYAs with *NF2*-SWN. These findings are concordant with the current surveillance recommendations of baseline brain MRI at age 10 years or time of diagnosis with annual ongoing surveillance frequency [5]. Spinal MRI surveillance begins at age 10 and is generally repeated every 3 years [5]. The clinical presentation of SWN in children and AYAs is often focal, and therefore localized imaging, rather than WB-MRI, is pursued. WB-MRI is often used to assess total tumor burden and identify lesions at risk for malignant conversion. In people with schwannomatosis, there is an extraordinarily low risk of malignant conversion. Furthermore, in younger children, surveillance imaging may involve sedation exposure and considerable logistical burden for caregivers, further emphasizing the clinical impact of these disorders beyond tumor prevalence alone.

The high tumor burden observed within this cohort also translated into considerable treatment complexity. A total of 48 tumor-related operative procedures were performed, including 19 spinal procedures (11 spinal schwannoma resections, seven spinal meningioma resections, and one ependymoma resection), nine cranial procedures (four VS debulking surgeries, four intracranial meningioma resections, and one orbital meningioma resection), and 20 peripheral nerve tumor procedures. Spinal surgery was most often performed for meningioma resections (7/7, 100%) with one instance of anaplastic WHO grade III meningioma and less frequently for spinal schwannoma (6/30, 20%). These findings highlight the significant morbidity associated with pediatric *NF2*-SWN and the need for interventions, even early in the disease course. In addition to operative management, many patients required non-operative management with bevacizumab as the most frequent treatment strategy. Among the various VS-directed therapies, bevacizumab has been most studied with emerging treatments including brigatinib, crizotinib, lapatinib and everolimus [27,28,29].

In contrast, non-*NF2* SWN subtypes were rare within this pediatric and AYA cohort and generally presented later, with longer diagnostic intervals, and with more limited tumor burden compared to those with *NF2*-SWN. This group comprised *LZTR1*-SWN, SWN-NOS, and SWN-NEC, including one patient with a positive family history. Prior studies including adult non-*NF2*-SWN populations have reported considerably older median ages at both initial clinical presentation (30 years) and diagnosis (40 years) [13]. Unlike *NF2*-SWN, no meningiomas or ependymomas were identified among patients with non-*NF2* SWN in this cohort [2,30]. Peripheral schwannomas were identified in two of three patients, lower than frequencies reported in adult non-*NF2*-SWN populations (89%) [13]. These findings likely reflect the evolving and age-dependent nature of SWN phenotypes. It is also possible that non-*NF2*-SWN remains under-recognized in younger populations.

Our cohort also highlighted several diagnostically challenging clinical scenarios, including a young patient with a solitary schwannoma, a symptomatic individual with a pathogenic *LZTR1* variant and imaging concerning a plexiform peripheral nerve tumor without pathological confirmation, and asymptomatic individuals with incidentally identified pathogenic variants associated with SWN.

Management of younger patients (<25 years) who present clinically with a solitary schwannoma remains challenging because of the concern regarding the possibility of SWN. Data from the United Kingdom suggests that 29% (44/153) of young patients with apparently isolated schwannomas ultimately meet criteria for SWN [31], although these data primarily involved intracranial and spinal schwannomas. Whether peripheral schwannomas carry a similar risk profile remains unclear. As such, multidisciplinary evaluation involving neurogenetics and/or genetic counseling, consideration of germline testing and longitudinal clinical follow-up may be beneficial in selected young patients presenting with solitary peripheral schwannomas. Whole-body MRI can play an important adjunctive role in this setting by identifying clinically occult schwannomas throughout the body that may alter diagnostic classification. However, optimal indications, timing, and surveillance paradigms for whole-body imaging in pediatric SWN-spectrum disorders remain undefined and warrant further study to inform robust guidelines.

Two additional individuals with incidentally identified pathogenic variants associated with SWN during evaluation for unrelated indications demonstrated no clinical or imaging evidence of schwannomas on advanced craniospinal imaging of SWN and were later diagnosed with Noonan syndrome [32,33]. These cases did not fulfill the clinical criteria of SWN. They are mentioned here because they illustrate the importance of pairing the clinical and molecular features to confirm an accurate diagnosis of SWN. At present, confirmation of a pathogenic germline variant alone, for any of the SWN forms other than *NF2*-SWN, does not satisfy diagnostic criteria for SWN. Advanced imaging techniques, including craniospinal and whole-body MRI, can detect clinically occult intracranial or intraspinal tumor burden and peripheral schwannomas in this context; however, there are no evidence-based guidelines, and concern about the cost–benefit of pursuing costly imaging for patients with incidentally detected pathogenic variants without a clinical phenotype.

Our study has several limitations, including its retrospective design and relatively small sample size, particularly among non-*NF2*-SWN subgroups. As a tertiary referral center cohort, the study population may additionally be biased toward more clinically severe disease, earlier diagnostic recognition, and higher imaging utilization compared with broader community populations. Histopathologic confirmation was not available for all lesions, and characteristic imaging features were therefore used as surrogate diagnostic markers in select cases. Despite the limitations, this study highlights the high imaging burden, complex diagnostic challenges, and multidisciplinary management needs associated with pediatric and AYA SWN-spectrum disorders in the era of modern molecular classification. Although genetic testing was available in 32/38 patients (84%), broader availability and utilization of molecular diagnostic advances may facilitate earlier and more accurate diagnosis, particularly in children and AYAs with atypical or incomplete clinical phenotypes. Continued longitudinal follow-up and larger multicenter pediatric cohorts with genetic confirmation will be essential to better define genotype–phenotype relationships, penetrance, natural history, and optimal imaging surveillance strategies in pediatric and AYA SWN.

## 5. Conclusions

In pediatric and AYA populations, *NF2*-SWN is the most common SWN subtype seen in a tertiary specialty center and is characterized by earlier clinical presentation, substantial tumor burden, and frequent need for multidisciplinary interventions. In contrast, non-*NF2*-SWN subtypes appear to be rare in younger populations, often presenting later and with more limited peripheral disease, although evolving penetrance and age-dependent tumor development likely contribute to under-recognition in childhood. Overall, these findings emphasize the importance of integrating molecular testing, advanced imaging, and multidisciplinary clinical evaluation in the assessment of pediatric and AYA patients with suspected SWN.

## Figures and Tables

**Table 1:** Demographic and clinical data in patients with *NF2*-related schwannomatosis.

			All NF2-SWN	De Novo Disease	Familial	*p*-Value
Number			31	24 (77%)	7 (23%)	
Sex		Male	9/31 (29%)	6/24 (25%)	3/7 (43%)	0.38
		Female	22/31 (71%)	18/24 (75%)	4/7 (57%)	
Median age at presentation in years (range in parentheses)			8 (0–18)	8 (2–18)	5 (0–16)	0.12
Duration to diagnosis (years)			0.5 (0–12)	0	0.75 (0–12)	0.006
Clinical presentation	Family history		5/31 (16%)	0	5/7 (72%)	<0.05
	Visual symptoms		6/31 (19%)	6/24 (25%)	0	0.29
	Hearing loss		4/31 (13%)	4/24 (17%)	0	1.0
	Soft tissue mass		5/31 (16%)	4/24 (17%)	1/7 (14%)	1.0
	Weakness		3/31 (10%)	3/24 (13%)	0	1.0
	Gait abnormality		3/31 (10%)	3/24 (13%)	0	1.0
	Seizure		2/31 (6%)	1/24 (4%)	1/7 (14%)	1.0
	Developmental delay		1/31 (3%)	1/24 (4%)	0	0.41
	Facial dysmorphism		1/31 (3%)	1/24 (4%)	0	1.0
	Dyskinesia		1/31 (3%)	1/24 (4%)	0	1.0
Diagnosed pre-symptomatically due to genetic testing			5/31 (16%)	0/24	5/7 (71%)	<0.05
Vision impairment at any time during follow-up			9/31 (29%)	7/24 (29%)	2/7 (29%)	1.0
Hearing loss at any time during follow-up			14/31 (45%)	11/24 (46%)	3/7 (43%)	1.0

Mann–Whitney U testing. Categories are not mutually exclusive.

**Table 2:** Comparison of age at presentation according to genetic severity score in patients with de novo and familial NF2-related schwannomatosis *.

Genetic Severity Group *	Median Age at Presentation in Years (Range)	Current Median Age in Years (Range)	All NF2-SWN **	De Novo Disease	Familial	*p*-Value
2A	4 (0–9)	13.5 (11–23)	4/19 (21%)	1/14 (7%)	3/5 (60%)	0.025
2B	9 (2–18)	20 (13–25)	7/19 (37%)	7/14 (50%)	0	
3	7.5 (3–18)	17 (10–21)	8/19 (42%)	6/14 (40%)	2/5 (40%)	

* In this cohort, there were no patients in GSS group 1 (mosaic pathogenic variants identified only in tumor tissue and not detected in blood). ** Among 25 patients with prior molecular testing, 19 had sufficient genetic data available for GSS classification.

**Table 3:** Imaging data and management in patients with *NF2*-related schwannomatosis.

		All NF2-SWN	De Novo Disease	Familial	*p*-Value *
Median * number of MRIs per patient (range in parentheses)	Total	23 (1–60)	24 (3–60)	22 (1–39)	0.22
	Brain and IACs	10 (1–36)	12 (2–36)	8 (1–25)	0.84
	Cervical spine	3 (1–7)	3 (1–7)	2 (1–4)	0.49
	Thoracic spine	1 (1–6)	2 (1–6)	1 (1–4)	0.50
	Lumbar spine	1 (1–24)	2 (1–24)	1 (1–1)	0.08
	Whole spine	1 (1–17)	5 (1–17)	5 (1–6)	0.57
	Whole body	1 (1–7)	1 (1–2)	7	0.86
	Chest/brachial plexus	2 (1–7)	2 (1–7)	0	0.35
	Pelvis/LS plexus	2 (1–9)	2 (1–9)	5	0.16
	Lower extremity	2 (1–6)	2 (1–6)	0	0.27
	Upper extremity	4 (2–4)	3 (2–4)	0	0.36
Bilateral vestibular schwannoma		30/31 (97%)	23/24 (96%)	7/7 (100%)	1.0
Non-vestibular cranial schwannoma		23/31 (74%)	17/24 (71%)	6/7 (86%)	0.64
Spinal schwannoma		30/31 (97%)	24/24 (100%)	6/7 (86%)	0.23
Cervical		21/31 (68%)	16/24 (67%)	5/7 (86%)	1.0
Thoracic		18/31 (58%)	14/24 (58%)	4/7 (57%)	1.0
Lumbar		24/31 (77%)	18/24 (75%)	6/7 (86%)	1.0
Peripheral schwannoma		19/31 (61%)	67/24 (33%)	3/7 (43%)	0.38
Cranial meningioma		19/31 (61%)	13/24 (54%)	6/7 (86%)	0.20
Spinal meningioma		7/31 (29%)	6/24 (25%)	1/7 (14%)	1.0
Cervical		5/31 (16%)	4/24 (16%)	1/7 (14%)	1.0
Thoracic		3/31 (10%)	3/24 (13%)	0	1.0
Ependymoma		11/31 (36%)	10/24 (42%)	1/7 (14%)	0.37
Cervical		11/31 (36%)	10/24 (42%)	1/7 (14%)	0.37
Thoracic		3/31 (10%)	2/24 (8%)	1/7 (14%)	0.55
Operative management		20/31 (65%)	16/24 (66%)	4/7 (47%)	1.0
Medical management		20/31 (65%)	16/24 (66%)	4/7 (57%)	1.0

* Mann–Whitney U testing.

**Table 4:** Spectrum of non-operative treatment strategies across patients with *NF2*-SWN.

Treatment Type	Primary Indication	Individuals, *n* (%)	Median Age (Range), Years
Any medical treatment		20/31 (65%)	21 (11–25)
Bevacizumab	Progressive VS	9/31 (29%)	22 (18–25)
Brigatinib	Progressive VS	8/31 (26%)	18 (11–23)
Gabapentin	Pain	6/31 (19%)	18 (15–25)
Levetiracetam	Seizure	4/31 (13%)	18 (13–21)
Crizotinib	Progressive VS	1/31 (3%)	21
Brivaracetam	Seizure	1/31 (3%)	21
Everolimus	Progressive VS	1/31 (3%)	23
Losartan	Proteinuria	1/31 (3%)	21

## Data Availability

The raw data supporting the conclusions of this article will be made available by the authors on request.
